# Gene losses did not stop the evolution of big brains

**DOI:** 10.7554/eLife.41912

**Published:** 2018-10-16

**Authors:** Cristian Cañestro, Vittoria Roncalli

**Affiliations:** 1 Departament de Genètica, Microbiologia i EstadísticaUniversitat de BarcelonaBarcelonaSpain; 2 Institut de Recerca de la Biodiversitat (IRBio)Universitat de BarcelonaBarcelonaSpain

**Keywords:** mammals, cetaceans, fruit bats, elephants, brain size, Other

## Abstract

Elephants and fruit bats have evolved large brains even though they have lost a gene that is fundamental to the supply of energy to the brain when glucose is not available.

**Related research article** Jebb D, Hiller M. 2018. Recurrent loss of *HMGCS2* shows that ketogenesis is not essential for the evolution of large mammalian brains. *eLife* **7**:e38906. doi: 10.7554/eLife.38906

The evolution of a big brain has been of long-standing interest due to its relationship to the origin of humans and its association with complex cognition (Darwin, 1874). Primates have brains that, relative to body size, are about twice as big as those of other mammals. The human brain has tripled in size over the last two million years (González-Forero and Gardner, 2018), and large brains have also evolved in many other mammalian species, including primates, dolphins, whales and elephants (Finarelli and Flynn, 2009; Fox et al., 2017; Goodman et al., 2009).

The evolution of a big brain, however, is very expensive in terms of energy: the brain of an adult human corresponds to just 2% of its weight, but accounts for 20% of the body's energy expenditure during rest. The situation is even more pronounced in newborns, with the brain accounting for about 10% of weight and 50% of energy consumption (Sokoloff, 1989). The evolution of metabolic systems for the production and storage of energy must, therefore, have limited the evolution of brain size. The fact that certain species often pass through periods of food shortage and fasting must also have influenced the evolution of brain size.

The primary source of energy for the brain is glucose, but when glucose levels drop, during fasting for example, the body starts to burn fatty acids instead. However, fatty acids are unable to cross the blood-brain barrier, so instead the brain relies on small water-soluble molecules called ketone bodies (which can cross the barrier) as its main source of energy. Ketone bodies are produced in the liver by a process called ketogenesis, which occurs in mitochondria and is catalyzed by an enzyme called *HMGCS2*. The raw material for the production of ketone bodies is a molecule called acetyl coenzyme (or acetyl-CoA for short), which in turn is derived from fatty acids. The ketone bodies are secreted into the blood by the liver and are transported to the brain and other organs that need energy.

It has been proposed, therefore, that in addition to having a crucial role during fasting, ketogenesis has also influenced the increase in brain size over the course of evolution (Wang et al., 2014). Now, in eLife, David Jebb and Michael Hiller – who are based at two Max Planck Institutes in Dresden (Molecular Cell Biology and Genetics, and the Physics of Complex Systems) – report the unexpected finding that big brains can evolve in mammalian species that are not capable of ketogenesis (Jebb and Hiller, 2018). This challenges the idea that this form of metabolism was a precondition for the evolution of a big brain.

By using a powerful whole-genome alignment approach (Sharma et al., 2018) in a collection of more than 70 mammalian genomes, Jebb and Hiller have discovered that the *HMGCS2* gene has been independently lost in three mammalian groups (Figure 1). These groups include species of cetaceans (e.g., dolphins and whales), proboscideans (e.g., elephants and extinct mastodons) and Old World fruit bats, and all of them have remarkably large brains and display signs of complex behaviors. The fact that Jebb and Hiller found that remnants of the *HMGCS2* gene within each group shared the same inactivating mutations has allowed them to date when the original inactivating mutation took place in each of the three groups. Interestingly, in the case of the cetaceans and proboscideans, the loss of the *HMGCS2* gene occurred before the period of brain expansion that affected dolphins and proboscideans (Figure 1).

**Figure 1.**
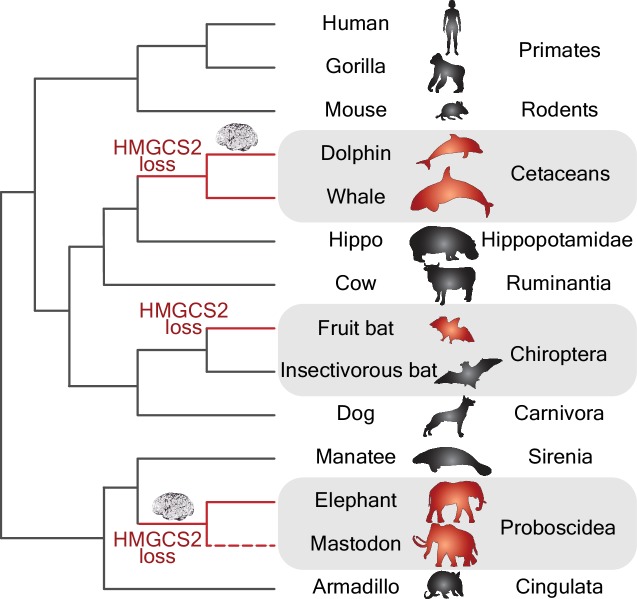
Gene losses and the evolution of big brains. Phylogenetic tree showing the independent losses of the *HMGCS2* gene in cetaceans (top grey region), fruit bats (middle grey region) and proboscideans (bottom grey region; the dashed line indicates that the mastodon is now extinct). The *HMGCS2* gene codes for the enzyme that is responsible for the production of ketone bodies, which are used by many mammals as a source of energy for the brain when glucose is not available. The results of Jebb and Hiller are surprising in that they suggest that the evolution of big brains (represented by the brain cartoons) in the lineages leading to dolphins and elephants might have occurred without ketone bodies being available as an energy source.

In addition to the loss of the *HMGCS2* gene, Jebb and Hiller checked for the loss of other ketogenic genes, and found that the *BDH1* gene, which codes for the enzyme that converts one ketone body (acetoacetate) into another (beta-hydroxybutyrate), had been also lost in the cetaceans and Old World fruit bats. This finding provides an example of the co-elimination of genes that are functionally linked in a distinct pathway (reviewed in Albalat and Cañestro, 2016). The apparent absence of a ketogenic pathway in these species led Jebb and Hiller to conclude that these animals might have acquired alternative fueling strategies during fasting.

The latest results raise a number of questions. How is it possible that an essential metabolic gene like the *HMGCS2* gene could be lost? And under what sort of evolutionary scenarios (that is, neutral or adaptive evolution) did these losses occur? Fruit bats have sugar-rich diet and do not have to worry about food because fruit is available throughout the year. It is plausible, therefore, that the gene loss was due to neutral evolution (that is, due to chance rather than natural selection) because the inability to produce ketone bodies offered no evolutionary disadvantage under such relaxed conditions. This possibility is supported by the observation that some fruit bats cannot resist starvation and die after less than 24 hours in captivity.

In the case of dolphins, glucose levels do not decrease, not even after long periods of fasting, thanks to a highly active metabolic pathway (the gluconeogenic pathway) that is capable of synthesizing glucose from amino acids that are abundant in their diet (Ridgway, 2013). It is plausible, therefore, that the loss of the *HMGCS2* gene in dolphins might also have been due to neutral evolution because the mutational robustness of this metabolic pathway means that, again, the inability to produce ketone bodies offered no evolutionary disadvantage.

This latest work by Jebb and Hiller, together with previous work from the same laboratory (Sharma et al., 2018), shows how crucial it is to discover events of gene loss in order to better understand the evolutionary genetic changes that have contributed to species diversification. Building up a complete catalogue of gene losses (which could be called a 'lossosome') in a phylogenetic context (Albalat and Cañestro, 2016) creates a framework that can be used to investigate how biodiversity has been generated. It is also a useful resource for understanding the biology of ecologically and economically important species, such as fruit bats, and effects of conservation efforts in support of such species.
